# The Variant rs1867277 in *FOXE1* Gene Confers Thyroid Cancer Susceptibility through the Recruitment of USF1/USF2 Transcription Factors

**DOI:** 10.1371/journal.pgen.1000637

**Published:** 2009-09-04

**Authors:** Iñigo Landa, Sergio Ruiz-Llorente, Cristina Montero-Conde, Lucía Inglada-Pérez, Francesca Schiavi, Susanna Leskelä, Guillermo Pita, Roger Milne, Javier Maravall, Ignacio Ramos, Víctor Andía, Paloma Rodríguez-Poyo, Antonino Jara-Albarrán, Amparo Meoro, Cristina del Peso, Luis Arribas, Pedro Iglesias, Javier Caballero, Joaquín Serrano, Antonio Picó, Francisco Pomares, Gabriel Giménez, Pedro López-Mondéjar, Roberto Castello, Isabella Merante-Boschin, Maria-Rosa Pelizzo, Didac Mauricio, Giuseppe Opocher, Cristina Rodríguez-Antona, Anna González-Neira, Xavier Matías-Guiu, Pilar Santisteban, Mercedes Robledo

**Affiliations:** 1 Hereditary Endocrine Cancer Group, Spanish National Cancer Research Centre (CNIO), Madrid, Spain; 2 Instituto de Investigaciones Biomédicas Alberto Sols, Consejo Superior de Investigaciones Científicas (CSIC), Autonomous University of Madrid (CSIC-UAM), Madrid, Spain; 3 ISCIII Centre for Biomedical Research on Rare Diseases (CIBERER), Madrid, Spain; 4 Familial Cancer Clinic, Veneto Institute of Oncology IRCCS, Padova, Italy; 5 Genotyping Unit-CEGEN, Spanish National Cancer Research Centre (CNIO), Madrid, Spain; 6 Genetic and Molecular Epidemiology Group, Spanish National Cancer Research Centre (CNIO), Madrid, Spain; 7 Hospital Universitario Arnau de Vilanova-IRB Lleida, Lleida, Spain; 8 Hospital de Requena, Valencia, Spain; 9 Hospital General Universitario Gregorio Marañón, Madrid, Spain; 10 Hospital Universitario Reina Sofía, Murcia, Spain; 11 Hospital Marina Alta, Denia, Spain; 12 Hospital General de Segovia, Segovia, Spain; 13 Hospital Reina Sofía, Córdoba, Spain; 14 Hospital General Universitario de Alicante, Alicante, Spain; 15 Hospital Universitario San Juan de Alicante, Alicante, Spain; 16 Hospital de Sabadell-Parc Taulí, Sabadell, Spain; 17 Hospital General Universitario de Elche, Elche, Spain; 18 Civil Hospital, Verona, Italy; 19 Surgical Pathology, Department of Medical and Surgical Sciences, University of Padova, Padova, Italy; 20 Department of Medical and Surgical Sciences, University of Padova, Padova, Italy; The University of Queensland, Australia

## Abstract

In order to identify genetic factors related to thyroid cancer susceptibility, we adopted a candidate gene approach. We studied tag- and putative functional SNPs in genes involved in thyroid cell differentiation and proliferation, and in genes found to be differentially expressed in thyroid carcinoma. A total of 768 SNPs in 97 genes were genotyped in a Spanish series of 615 cases and 525 controls, the former comprising the largest collection of patients with this pathology from a single population studied to date. SNPs in an LD block spanning the entire *FOXE1* gene showed the strongest evidence of association with papillary thyroid carcinoma susceptibility. This association was validated in a second stage of the study that included an independent Italian series of 482 patients and 532 controls. The strongest association results were observed for rs1867277 (OR[per-allele] = 1.49; 95%CI = 1.30–1.70; *P* = 5.9×10^−9^). Functional assays of rs1867277 (NM_004473.3:c.−283G>A) within the *FOXE1* 5′ UTR suggested that this variant affects *FOXE1* transcription. DNA-binding assays demonstrated that, exclusively, the sequence containing the A allele recruited the USF1/USF2 transcription factors, while both alleles formed a complex in which DREAM/CREB/αCREM participated. Transfection studies showed an allele-dependent transcriptional regulation of *FOXE1*. We propose a *FOXE1* regulation model dependent on the rs1867277 genotype, indicating that this SNP is a causal variant in thyroid cancer susceptibility. Our results constitute the first functional explanation for an association identified by a GWAS and thereby elucidate a mechanism of thyroid cancer susceptibility. They also attest to the efficacy of candidate gene approaches in the GWAS era.

## Author Summary

Although follicular cell-derived thyroid cancer has an important genetic component, efforts in identifying major susceptibility genes have not been successful. Probably this is due to the complex nature of this disease that involves both genetic and environmental factors, as well as the interaction between them, which could be ultimately modulating the individual susceptibility. In this study, focused on genes carefully selected by their biological relation with the disease, and using more than 1,000 cases and 1,000 representative controls from two independent Caucasian populations, we demonstrate that *FOXE1* is associated with Papillary Thyroid Cancer susceptibility. Functional assays prove that rs1867277 behaves as a genetic causal variant that regulates *FOXE1* expression through a complex transcription factor network. This approach constitutes a successful approximation to define thyroid cancer risk genes related to individual susceptibility, and identifies FOXE1 as a key factor for its development.

## Introduction

Thyroid cancer is the most common endocrine malignancy, and accounts for 1% of all neoplasias [1]. Among them, papillary thyroid carcinoma (PTC, 80–85 % of cases), and follicular thyroid carcinoma (FTC, 5–10 %) are the most frequent [2]. The etiology of PTC, both sporadic (95 % of cases) and familial (about 5 %), seems to be rather complex. Exposure to ionizing radiation and deficiency in iodine intake have been suggested as environmental risk factors related to PTC and FTC, respectively [3]. Different genetic alterations involving the RET/PTC-RAS-BRAF signalling pathway have been described as causal somatic changes in PTC and FTC [4]–[9]. In addition, PTC has a strong genetic component, since it shows one of the highest relative risks (FRR = 8.60–10.30) in first degree relatives of probands among cancers not displaying Mendelian inheritance [10],[11]. Several putative loci associated with familial forms of PTC have been suggested by linkage analysis [12]–[15], although no high penetrance gene has been convincingly described, even within the putative loci, probably due to the heterogeneity of the disease. Finally, microRNAs (miRs) have also been suggested to be involved in the disease [16],[17], although their specific role remains unclear.

Therefore, it is expected that thyroid cancer is the result of multiple low- to moderate-penetrance genes (LPGs) interacting with each other and with the environment, thus modulating individual susceptibility [10],[18]. In this scenario, linkage analysis does not have the power to identify these LPGs [19],[20]. Thus, GWAS or carefully designed candidate gene approaches may be more appropriate strategies to define genetic risk factors.

We performed a candidate gene association study in thyroid cancer, showing that *FOXE1*, formerly called *TTF2* (Thyroid Transcription Factor 2), exhibits the strongest association with PTC susceptibility. *FOXE1* itself is a good candidate LPG because it is the centre of a regulatory network of transcription factors and cofactors that initiate thyroid differentiation [21] and whose function is essential for thyroid gland formation and migration, as well as for the maintenance of the thyroid differentiated state in adults [22].

Our study, which involves the largest collection of patients with this pathology from a single population to be studied to date, also identifies a causal variant within *FOXE1* as well as the underlying molecular mechanism involved. The variant rs1867277 (NM_004473.3:c.−283G>A) within the *FOXE1* 5′ UTR affects gene transcription through differential recruitment of USF1/USF2 transcription factors only when the −283A allele is present. By contrast, a protein complex in which DRE- and CRE-binding proteins participate, binds to both alleles.

Recently, a genome-wide association study (GWAS) identified two SNPs located at 9q22.23 and 14q13.3 that are strongly associated with an increased risk of PTC and FTC [23]. The closest gene to the top marker at 9q22.33, rs965513 (OR = 1.75; *P* = 1.7×10^−27^) is *FOXE1*.

Overall, it is noteworthy that, in this particular case, both strategies identify the same gene, although the study of carefully selected candidate genes remains a more direct, practical and efficient approach to reveal functional variants within LPGs.

## Methods

### Subjects

Patients diagnosed with thyroid cancer were recruited from the Spanish hospital network. A total number of 615 cases were available for the study, representing, to our knowledge, the largest thyroid cancer series from a single population. Our series included the main thyroid follicular-cell derived carcinomas: 520 PTC, represented by their main subtypes ‘classic PTC’ (cPTC; n = 304) and ‘follicular variant PTC’ (FVPTC; n = 146), as well as 69 Follicular Thyroid Carcinomas (FTC). Medullary Thyroid Carcinomas (MTC) were not included in the study, since we previously performed a similar study of these cases [24]. Clinicians fulfilled a detailed clinical questionnaire for all patients, which included questions regarding both personal and clinical data, such as tumour subtype and stage, surgery option, treatment details in terms of ^131^I doses, and development of metastasis during the follow-up. Diagnoses were assessed by pathologists from the different institutions that participated in the study.

A series of 525 healthy controls, free of cancer and representative of the Spanish population, were selected as the reference group. These subjects came from the same geographical regions as covered by the hospitals involved in the study. Informed consent was obtained from all subjects included in the study.

Median age and sex ratio (female∶male) were 46 years and 4.6, respectively, in both cases and controls. Mean age and gender distribution were similar in controls and cases (Mann-Whitney's U and Kruskal-Wallis associated *Ps*>0.05).

An additional 78 Spanish cases, obtained over the last year, were genotyped for the significant SNP analyzed in most detail, in order to increase the power of the test. These additional patients were recruited from the same hospitals as the original Spanish series. The distributions of thyroid cancer subtype, age and gender were also similar to those of the first series (*P*>0.05).

A second stage of the study consisted of an independent Italian series, used as a validation set, and was composed of 482 thyroid cancer patients and 532 representative controls. Cases included 412 individuals with PTC and 44 with FTC. Their median age at diagnosis and female∶male sex ratio were 48.5 years and 4.9, respectively. Italian controls were recruited from the same two geographical regions as cases, and had a similar sex ratio and age distribution as cases.

The design of the study is summarized in Figure S1.

### DNA isolation and quantification

Blood (n = 878) or saliva (n = 262) samples were obtained from Spanish patients and controls. Genomic DNA was extracted from peripheral blood lymphocytes by automated DNA extraction according to the manufacturer's instructions (Magnapure, Roche) and from saliva using the Oragene DNA Self-Collection Kit (DNA Genotek, Ottawa, Canada). Genomic DNA was isolated from Italian blood samples (n = 1014) using standard methods [25].

DNA concentration was quantified in all samples prior to genotyping by using Quant-iT PicoGreen dsDNA Reagent (Invitrogen, Eugene, OR, USA).

### Gene selection

We used a candidate gene approach in this study. Three different criteria were used for selecting loci. First, we chose genes we found to be differentially expressed in primary thyroid tumours and normal tissue [26], or as described in public databases, such as CGAP-SAGE (http://cgap.nci.nih.gov/SAGE). Second, we picked genes involved in thyroid follicular cell biology and metabolism. Finally, critical metabolic pathways such as the MAP kinase, JAK-STAT and TGF-beta signaling pathways were represented by selecting genes encoding proteins that play key roles in those pathways (membrane receptors, signal transducers, transcription factors, inhibitors, etc.). The latter criterion was applied after an exhaustive review of the information contained in the pathway databases KEGG Pathways (http://www.genome.ad.jp/kegg/pathway.html), Biocarta (http://www.biocarta.com/genes/index.asp), and Pathway Studio 4.0 (evaluation version). Candidate non-coding MIRN genes were considered in the initial list using miRBase (http://microrna.sanger.ac.uk/sequences/).

We ranked the *loci* based on the above criteria and finally selected 97 genes for our association study (manuscript in preparation).

### SNP selection

The selected genes were represented by Single Nucleotide Polymorphisms (SNPs) within the intragenic region and within the regions spanning 10 kilobases upstream (to cover the hypothetical entire promoter area) and 2 kilobases downstream of the gene. We chose a total number of 768 SNPs, that can be divided into two main categories: (i) 523 ‘tag SNPs’, used to infer Linkage Disequilibrium (LD) blocks according the HapMap project (http://www.hapmap.org/) [27]; and (ii) 245 potentially functional SNPs, as predicted by bioinformatic tools PupaSuite (http://pupasuite.bioinfo.cipf.es/) [28] and F-SNP (http://compbio.cs.queensu.ca/F-SNP/) [29]. Predictions of functionality included SNPs that caused an aminoacid change in the protein (non-synonymous SNPs), as well as those variants located within putative transcription factor binding sites (TFBS) and exonic splicing enhancers (ESE). SNP codes, locations, and frequencies were obtained from the NCBI SNP database, build 126 (http://www.ncbi.nlm.nih.gov/projects/SNP/).

The initial list of more than 52,000 SNPs fulfilling the above criteria was filtered by applying the following additional criteria: (i) a threshold minor allele frequency (MAF) in the HapMap-CEU population of 0.10 for ‘tag SNPs’ and of 0.02 for putative functional SNPs; and (ii) an ‘Illumina score’ not less than 0.6 (as recommended by the manufacturer), to ensure a high genotyping success rate. No variants within the MIRN genes considered were described in the databases. Finally, we selected and genotyped 768 SNPs within 97 loci, thus fulfilling the platform requirements.

### SNP genotyping

SNPs were genotyped using the Illumina GoldenGate Genotyping Assay (San Diego, CA, USA) system, on a Sentrix Universal-96 Array Matrix multi-sample array format. Genotyping was carried out using 400 nanograms of DNA per reaction following the manufacturer's instructions (http://www.illumina.com/). Genotyping specificity was assessed by including two DNA duplicates (an intra-assay and an inter-assay duplicate) and a negative control in each 96-well plate genotyped, yielding 100% consistent replication results. In addition, cases and control samples were always included in the same run.

Validation set genotyping was performed by means of the KASPar SNP Genotyping System (Kbiosciences, Herts, UK). Fifteen nanograms of DNA were used for the genotyping reactions. The 7900HT Sequence Detection System (Applied Biosystems, Foster City, CA, USA) was used for fluorescence detection and allele assignment.

An additional variant, rs1867277, not included in the Illumina assay, was selected for its predicted effect on the transcriptional activity of *FOXE1* to perform functional assays. This SNP was analysed on a subset of 200 cases and controls by DHPLC on the WAVE HT system (Transgenomic, Omaha, NE) using an acetonitrile gradient; it was scrutinised for aberrant profiles with the Navigator software (Transgenomic, Omaha, NE) to determine the correlation between this potential functional SNP and the other variants in *FOXE1* genotyped by the Illumina platform.

Genotyping accuracy of both KASPar and DHPLC technologies was confirmed by direct sequencing of 5% of the samples selected at random.

### Cell cultures and plasmids

WRO cells derived from a human follicular thyroid carcinoma were cultured as described [30]. The expression vectors used were pcDNA3.1-DREAM [31], pSG5-αCREM [32], pGal4-CREB [33], pN3 (USF1), pN4 (USF2) [34] and pUSF-1, expressing a dominant negative form of USF1. The pGl3b-FOXE1 reporter construct contains the 5′ upstream regulatory region from −1934 to +539 bp relative to the transcription start site of human *FOXE1* [35] and carries an A allele for the rs1867277 SNP. In this plasmid, the A allele was replaced by the G allele (pGl3b-FOXE1-283G) by means of site directed mutagenesis (QuikChange II XL kit; Stratagene) following the manufacturer's instructions using the oligonucleotide 5′-cagtcccggtc[g]cgaggccaccgc-3′. The c.−283A>G substitution and the absence of artefacts were confirmed by direct sequencing. The vector pRL-CMV, containing a cDNA coding for *Renilla*, was used to monitor transfection efficiency.

### Electrophoretic mobility shift assays

Nuclear protein extracts from WRO cells were obtained following standard procedures [36]. Specific proteins were synthesized from the USF1/2 expression vectors pN3 and pN4 by *in vitro* transcription/translation using the TNT coupled reticulocyte lysate system (Promega).

Seven µg of protein extracts or 3 µl of TNT pools were incubated with 200 ng of the corresponding dsDNA probes representing rs1867277-A: 5′-gtcccggtcAcgaggccaccg-3′ (referred to as “Allele A”); rs1867277-G: 5′- gtcccggtcGcgaggccaccg-3′ (“Allele G”); and the DRE element from the prodynorphin gene: 5′- gaagccggagtcaaggaggcccctg-3′ (“DRE-pDyn”), previously labelled with γ^32^-ATP by T4 polynucleotide kinase. For competition, a 100-fold excess of the same (“related”) or different (“unrelated”: 5′-ccataatgcaaaaatggaaagaattaaa-3′) unlabeled oligonucleotide was used as indicated in each experiment. Additional dsDNA probes used were: the USF consensus sequence (USF-cons) 5′-cctgcccacgtgacccggcct-3′; the CRE binding region of the somatostatin gene (CRE-Cons) 5′-cctcctagcctgacgtcagagagagagt-3′; and the CRE-like region of the *FOXE1* gene (CRE-FOXE1) 5′-accagagtcgagtcccggtcacgaggcca-3′. When required, specific antibodies recognizing human DREAM, USF1, or USF2 (sc-9142, sc-229 and sc-862, respectively, from Santa Cruz Biotechnologies) were incubated together with protein extracts and dsDNAs. EMSA conditions were similar to those described previously [24].

### Transfection assays

Hela cells were transient transfected using the JetPei reagent (PolyPlus Transfection) with different amounts of expression vector (as indicated in each experiment); 3 µg of reporter construct and 60 ng of pRL-CMV were used. Forty-eight hours after transfection, cells were harvested, lysed, and analyzed for luciferase and Renilla activities. The promoter activity in cells transfected with the expression vector was determined as the ratio between luciferase and Renilla, relative to the ratio obtained in cells transfected with the corresponding empty expression vector. The results shown are the average±SD of three independent experiments, each performed in triplicate.

### Statistical analysis

Departure from Hardy-Weinberg equilibrium (HWE) for all SNPs was tested in controls using Fisher's exact test. Associations between each SNP and thyroid cancer risk were assessed using Pearson's χ^2^ test. Genotype frequencies in cases and controls were compared and odds ratios (OR) per allele were estimated by applying unconditional logistic regression, using homozygotes of the most frequent allele in controls as the reference group. Significant *P* values were adjusted for the putative confounding factors age, gender, and population.

Haplotypes were inferred using PHASE 2.0, a computational tool based on Bayesian methods. Case-control comparisons of haplotype distributions were carried out by applying the inbuilt permutation test, based on 10,000 permutations. Associations between specific haplotypes and risk of thyroid cancer were assessed using the Haplo.Stats package in R (http://www.R-project.org).

For transfection assays, statistical significance was determined by t-test analysis (two-tailed), and differences were considered significant with *P*<0.05.

Statistical tests were performed using SPSS for Windows 17.0 software.

## Results

Polymorphisms within the *FOXE1* locus are significantly associated with Papillary Thyroid Carcinoma in the Spanish population

From the initial list of 768 variants, 33 SNPs (4 %) were discarded due to a low fluorescence signal. Forty-five additional polymorphisms (6 %) did not fulfill Hardy-Weinberg equilibrium (*P*<0.05) in control samples and were excluded from the following analyses. Overall, 690 SNPs (90 %) were studied. Among them, top association results in the Spanish population were observed for SNPs located within the *FOXE1* locus (for the best tagSNP, OR[per-allele] = 1.47; 95% CI = 1.23–1.75; *P* = 2.4×10^−5^), strongly suggesting a putative LPG role for this gene, specifically in the PTC subtype. Phase I association results are summarized in Table 1. It is worthy to note that, when stratifying according to the different PTC histological subtypes, a stronger association was detected for *FOXE1* SNPs exclusively for ‘classic PTC’ (Table S1).

**Table 1:** Significant polymorphisms in the *FOXE1* locus when comparing Papillary Thyroid Cancer patients against controls in the Spanish population (Phase I).

SNP name	Coordinate	Location	Function/ prediction	Alleles (major/minor)	Genotyped samples (controls/cases)	MAF in controls	MAF in cases	OR^a^ (95% CI)	*P*
rs7048394	99645254	Upstream	tagSNP	C/T	504/520	0.243	0.315	1.46 (1.19–1.78)	2.4×10^−4^
rs894673	99652091	Upstream	tagSNP	T/A	504/520	0.400	0.482	1.39 (1.17–1.65)	2.2×10^−4^
rs3758249	99653961	Upstream	TFBS	G/A	503/517	0.399	0.483	1.39 (1.17–1.66)	1.9×10^−4^
rs907577	99654938	Upstream	TFBS	A/G	503/518	0.399	0.482	1.39 (1.17–1.65)	2.1×10^−4^
rs3021526	99656842	Exon 1	S275S	T/C	495/475	0.398	0.469	1.32 (1.11–1.58)	2.1×10^−3^
rs10119760	99664423	Downstream	tagSNP	C/G	504/518	0.344	0.437	1.47 (1.23–1.75)	2.4×10^−5^

a OR [per allele] obtained by comparing cases against controls and considering the minor allele as the risk allele.

MAF, Minor Allele Frequency; OR, Odds Ratio; CI, Confidence Interval; TFBS, Transcription Factor Binding Site.

### Haplotype analysis identifies a highly significant risk haplotype specifically associated with classic PTC cases

**Figure 1:**
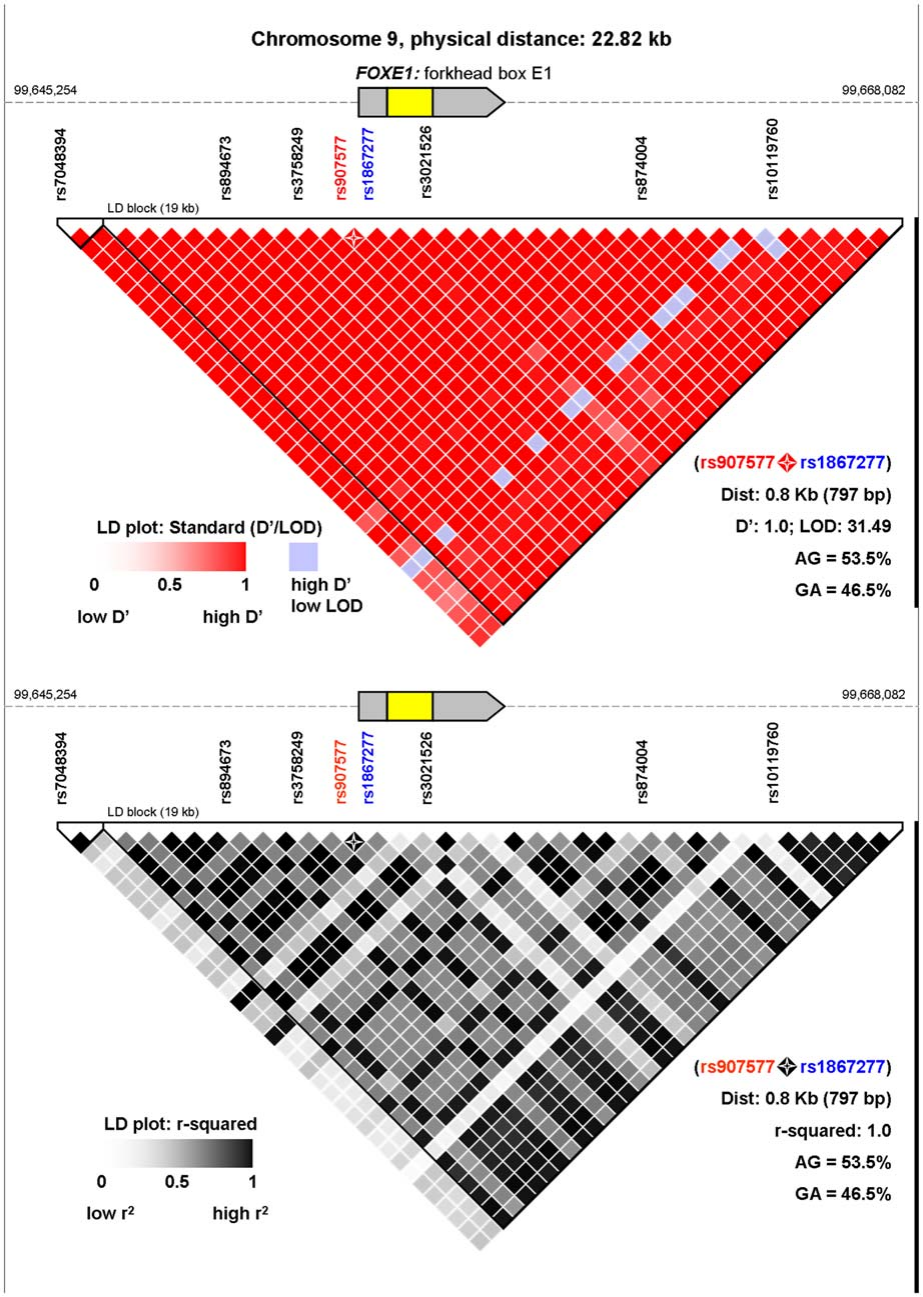
LD plot representation of the *FOXE1* locus. The figure represents linkage disequilibrium based on the HapMap-CEU population data (Data Release 24/phase II Nov08, on NCBI B36 assembly, dbSNP b126) along the *FOXE1* locus. The region spans chromosome 9 from coordinates 99,645,254 to 99,668,082 for a total length of 22.82 kilobases. The *FOXE1* gene is represented in yellow (exon) and grey (5′ and 3′ untranslated regions). LD is measured by D'/LOD parameters (top panel) and the r-squared correlation value (bottom panel). D' and r-squared values of 1 mean complete LD. The region is tagged by the SNPs shown in the figure, all of which were highly significant in the phase I association study. The functional variant rs1867277 is shown in blue, and the tagSNP rs907577 is shown in red. Specific LD values between these two variants are shown in the figure (four-point star).

Five out of six significant SNPs in *FOXE1* were located in a single LD block (Figure 1), and were subsequently used for haplotype analysis of the region spanning from chromosomal coordinates 99,648,503 to 99,668,059 (Figure 1, Table 2). This approach, used to reinforce results from individual SNP studies, showed a significant difference in haplotype distribution between all PTC cases and controls (PHASE associated *P* = 0.0147). Again, these differences were stronger when considering the classic PTC subtype (PHASE associated *P* = 0.0005, Table S2). Among the ten haplotypes inferred in our population, three represented 95% of individuals. The remaining seven haplotypes showed frequencies lower than 1%, and were not considered for further analyses. A detailed study of the three mentioned *FOXE1* haplotypes (shown in Table 2), allowed us to identify a risk haplotype significantly overrepresented in classic PTC patients (OR = 1.66; *P* = 0.0005).

**Table 2:** Haplotype analysis of the *FOXE1* locus in Spanish classic Papillary Thyroid Carcinoma cases versus controls.

Haplotype ID	Haplotype frequencies^a^		SNP ID (chromosomal coordinate)^b^							Haplotype associated risks^c^	
	Controls	Cases (classic PTC)	rs894673 (99,652,091)	rs3758249 (99,653,961)	rs907577 (99,654,938)	rs1867277 (99,655,735)	rs3021526 (99,656,842)	rs874004 (99,661,939)	rs10119760 (99,664,423)	OR	*P*
**Haplotype #1 >**	0.26	0.19	T	G	A	G	T	C	C	1	
**Haplotype #2 >**	**0.34**	**0.44**	**A**	**A**	**G**	**A**	**C**	**C**	**G**	**1.66**	**0.0005**
**Haplotype #3 >**	0.34	0.32	T	G	A	G	T	G	C	1.25	0.1355

a Haplotype frequencies were estimated using the PHASE program with 10,000 permutations.

b The LD region studied spans from coordinates 99,648,503 to 99,668,059 on chromosome 9 for a total length of 19.5 kb.

c Haplotype risks were calculated using the Haplo.Stats package in R, considering the overrepresented haplotype in controls (haplotype #1) as the reference.

Significant risk.conferring haplotype (haplotype #2) is highlighted in bold case.

PTC, Papillary Thyroid Carcinoma; OR, Odds Ratio.

### A detailed analysis of the PTC-risk conferring haplotype identifies rs1867277 as a highly correlated putative functional variant within the *FOXE1* promoter

Since none of the six *FOXE1*-associated variants showed a consistent putative functional role (according to the bioinformatics tools used), a more detailed analysis of the sequence across this LD region was performed. This allowed us to identify a functionally interesting SNP (rs1867277, -283G>A), not initially included in the Illumina assay. According to bioinformatics predictions, this variant may influence the binding of transcription factors that could regulate *FOXE1* transcription. DHPLC results in a subset of 200 individuals allowed us to experimentally confirm a complete LD between this functional variant (rs1867277) and the rs907577 tagSNP (included in the Illumina assay), and thus to impute genotypes of the first variant (OR[per allele] = 1.39; *P* = 2.1×10^−4^). This correlation is represented in Figure 1, and specific LD values are provided in Table S3.

Interestingly, the risk haplotype identified (Table 2, haplotype #2), included the rs907577 G allele and the functional variant rs1867277 A (located only 797 bases downstream), which has an effect on *FOXE1* transcription (see below).

### Phase II association study validates *FOXE1* rs1867277 in Papillary Thyroid Cancer

An independent Italian population (phase II) validated the phase I association results for the rs1867277 *FOXE1* promoter variant. This polymorphism was found to be significantly overrepresented in a series of 405 Italian PTC cases *versus* 525 Italian controls (OR[per allele] = 1.64; 95% CI = 1.31–2.05; adjusted *P* = 1.3×10^−5^). Phase I, II, and combined analyses for both series are summarized in Table 3. Pool analysis for rs1867277 in 984 PTC cases *vs.* 1028 controls confirmed the involvement of this *FOXE1* variant in PTC development (OR[per allele] = 1.49; 95% CI = 1.30–1.70; adjusted *P* = 5.9×10^−9^).

**Table 3:** Combined association results for *FOXE1* promoter variant rs1867277 (c.−283 G>A) in Papillary Thyroid Carcinoma patients against controls.

Genotyped samples	rs1867277 A frequency		Unadjusted results				Adjusted results^g^	
(n PTC/ n controls)	PTC	Controls	OR het^d^ (95% CI)	OR hom^e^ (95% CI)	OR per allele (95% CI)	*P* f	OR per allele (95% CI)	*P* f
Spain (518/503)^a^	0.482	0.399	1.46 (1.10–1.94)	1.90 (1.34–2.71)	1.39 (1.17–1.65)	2.1×10^−4^	1.38 (1.15–1.64)	3.9×10^−4^
Spain (579^b^/503)	0.484	0.399	1.50 (1.14–1.97)	1.95 (1.38–2.74)	1.41 (1.19–1.67)	8.5×10^−5^	1.39 (1.17–1.65)	1.8×10^−4^
Italy (405/525)^c^	0.558	0.456	1.64 (1.18–2.30)	2.38 (1.62–3.50)	1.54 (1.27–1.87)	9.9×10^−6^	1.64 (1.31–2.05)	1.3×10^−5^
Spain+Italy (984/1028)	0.515	0.428	1.48 (1.20–1.83)	2.01 (1.56–2.59)	1.42 (1.25–1.61)	4.2×10^−8^	1.49 (1.30–1.70)	5.9×10^−9^

a rs1867277 genotypes inferred from adjacent tagSNP rs907577, after proving a total Linkage Disequilibrium between both variants (see body text).

b An additional 61 Spanish PTC genotyped for rs1867277 by means of KASPar technology.

c Validation series genotyped for rs1867277 by means of KASPar probes.

d OR heterozygous: GG *vs*. AG genotypes.

e OR homozygous: GG *vs*. AA genotypes.

f *P* values are derived from ORs [per allele].

g Adjusted for age, gender, and origin.

PTC, Papillary Thyroid Carcinoma; OR, Odds Ratio; CI, Confidence Interval.

### The transcription factor DREAM binds within the *FOXE1* promoter at the region containing the rs1867277 risk variant

**Figure 2:**
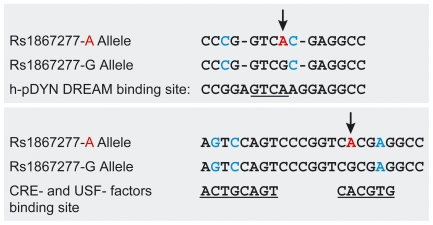
Sequence analysis of rs1867277 in the *FOXE1* promoter. (Top Panel) The A risk allele is shown in red and marked with an arrow. Nucleotides in blue indicate mismatches with regard to the DREAM consensus sequence, and gaps (indicated by dashes) are introduced into the sequences of both alleles to optimize alignment with the DREAM binding site. The DRE site derived from the human prodynorphin (h-pDYN) promoter is underlined. (Bottom Panel) Alignment of sequences close to the A and G alleles; consensus CRE- and USF-transcription factor binding sites are underlined. Bases in blue indicate differences with the consensus sequences.

**Figure 3:**
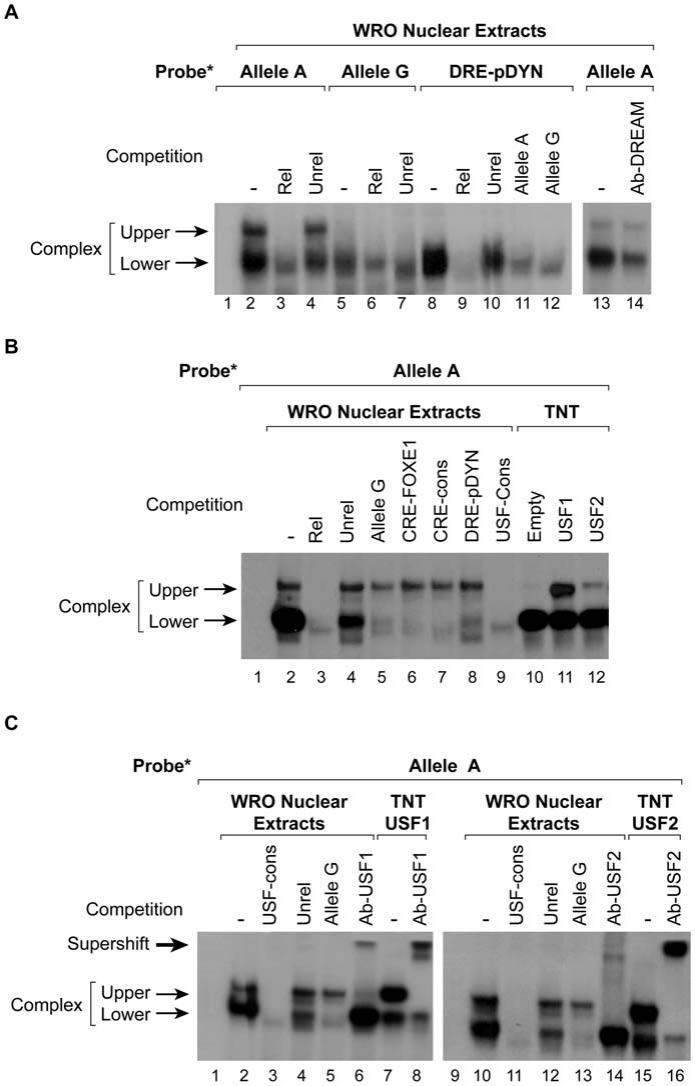
EMSAs show that different nuclear proteins bind to the *FOXE1* rs1867277 region. (A) Nuclear extracts were prepared from WRO cells for EMSA assays with ^32^P-labeled oligonucleotides corresponding to rs1867277 A, rs1867277 G, or the consensus DRE derived from the prodynorphin promoter (DRE-pDYN). The probes were incubated without (lane 1) or with nuclear extracts (lanes 2 to 14). Competition was performed with a 100-fold molar excess of unlabeled related (lanes 3, 6, 9), unrelated (lanes 4, 7, 10,) or alleles A and G (lanes 11 and 12) oligonucleotides, as well as with an anti-DREAM antibody (lane 14). Protein/DNA complexes are indicated by arrows. (B) A ^32^P-labeled probe containing the oligonucleotide corresponding to the rs1867277 A allele was incubated alone (lane 1); with WRO nuclear extracts (lanes 2-9); or with TNT translated proteins from empty vector (lane 10), or from USF1 or USF2 expression vectors (lanes 11, 12). Competition was performed with a 100-fold molar excess of unlabeled related (lane 3), unrelated (lane 4), allele G (lane 5), CRE-FOXE1 (lane 6), CRE consensus (lane 7), DRE-pDYN (lane 8), or USF consensus (lane 9) oligonucleotides. (C) A ^32^P-labeled probe containing the oligonucleotide corresponding to the rs1867277 A allele was incubated alone (lanes 1, 9); with WRO nuclear extracts (lanes 2–6 and 10–14); or with TNT-translated USF1 (lanes 7, 8) or USF2 protein (lanes 15, 16). Competition was performed with a 100-fold molar excess of unlabeled USF consensus (lanes 3 and 11), unrelated (lanes 4 and 12), or allele G (lanes 5 and 13) oligonucleotides. In addition, supershift assays were performed with specific anti-USF1 (lanes 6 and 8) or anti-USF2 (lanes 14 and 16) antibodies. Protein/DNA complexes are indicated by arrows. The amount of proteins incubated with the probes was 7 µg of WRO nuclear extracts and 3 µl of TNT reaction for in vitro translated proteins.

rs1867277 lies within a sequence with high similarity to the DRE consensus core sequence described for the prodynorphin promoter region [37] (Figure 2). In a first attempt to evaluate the role of rs1867277 in the transcriptional regulation of *FOXE1*, nuclear extracts from WRO cells were tested for their ability to bind both A and G alleles in an EMSA assay. As shown in Figure 3A, protein/DNA complexes were differentially formed when using A and G-allele probes (lanes 2 and 5, respectively): a lower complex was formed with both alleles, while an upper complex was formed exclusively with the A allele. The complexes were specific, as they were competed by a 100-fold excess of unlabelled related oligonucleotide (lanes 3 and 6) but not by an unrelated one (lanes 4 and 7).

In order to identify the transcription factors that bind to both alleles, we first focused on DREAM (Downstream Regulatory Element Antagonist Modulator) since rs1867277 contains a putative DRE consensus sequence (Figure 2). When the prodynorphin promoter containing the DRE consensus sequence (DRE-pDYN) was used as a probe, a specific complex with the same mobility as the A or G-allele lower band was detected (Figure 3A, lane 8; related and unrelated competition, lanes 9 and 10). Interestingly, this complex was partially competed by A and G-allele oligonucleotides (lanes 11 and 12). Furthermore, a specific DREAM antibody substantially reduced the intensity of the lower band (lane 14). These data demonstrate the involvement of endogenous DREAM in the lower shifted complex, although the lack of total competition when oligonucleotides containing the A or G allele were used, suggests that other proteins may be a part of this complex. Intriguingly, a CRE-like sequence was identified close to rs1867277 (Figure 2); CRE-binding factors can behave as interacting partners of DREAM [38].

### USF proteins bind specifically to the rs1867277 A allele

As the upper complex was exclusively formed when the A allele was present, we decided to identify the transcription factor/s that form part of that complex by interrogating databases (Gene Regulation, http://www.gene-regulation.com). The Upstream Stimulatory Factors (USF1 and USF2) were predicted to bind exclusively to the rs1867277 A allele.

Thus, EMSAs with the A allele were performed with different oligonucleotides as competitors, including a USF consensus sequence and several oligonucleotides containing CRE sequences, due to the relationship between DRE- and CRE (Figure 3B). When the oligonucleotide containing the G allele was used, only the lower complex was competed, demonstrating the specificity of the upper complex for the A allele (lane 5). Similarly, CRE-like FOXE1, the consensus CRE sequence, and consensus DRE-pDyn competed exclusively with the lower band (lanes 6–8), suggesting that the lower complex contains more than one transcription factor. Interestingly, the upper band was totally competed when the USF consensus sequence was used (lane 9). Furthermore, the *in vitro* translated (TNT) USF1 or USF2 proteins formed a complex with a similar mobility as the upper complex (lanes 11–12). When specific antibodies against either transcription factor were added, the upper complex formed over the A allele was supershifted when using nuclear extracts (Figure 3C, lanes 6 and 14), and also when using *in vitro* translated USF proteins (lanes 8 and 16). This unequivocally demonstrates that USF1/USF2 form part of the upper complex. On the other hand, the rs1867277 G allele shift and supershift experiments using the same approach did not show this upper DNA-protein complex (not shown).

Altogether, these results strongly indicate that only the rs1867277 A allele is able to form a protein complex that includes the transcription factors USF1 and USF2. In addition, both rs1867277 A and G form a complex in which DREAM and possibly other CRE-binding related transcription factors participate.

### USF1 and USF2 regulate transcriptional activity of the *FOXE1* promoter

**Figure 4:**
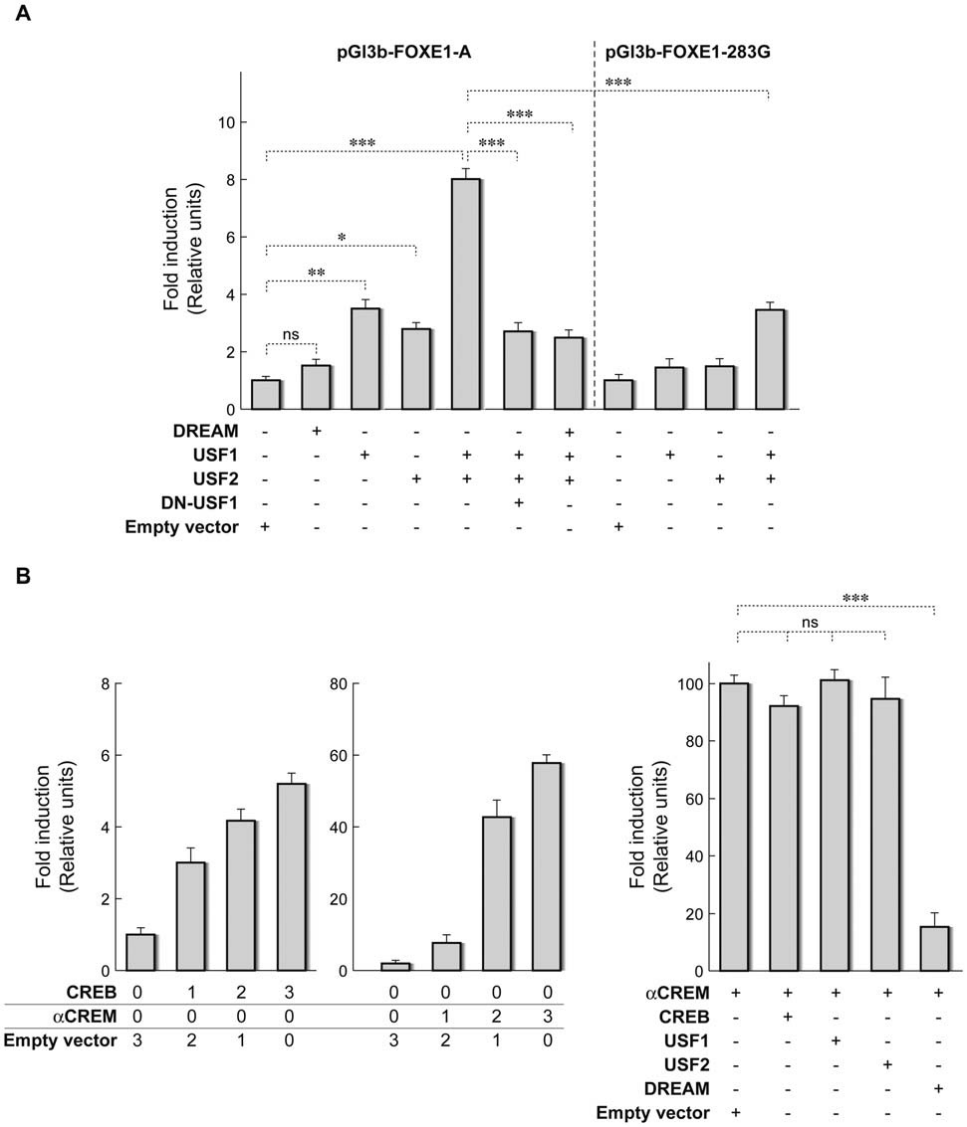
Transcriptional activity of the *FOXE1* promoter. (A) The *FOXE1* promoter containing the A allele (pGl3b-FOXE1-A) or the G allele (pGl3b-FOXE1-283G) was cotransfected into HeLa cells with 3 µg of the empty expression vector, or with 3 µg of the vector harbouring the cDNAs for DREAM, USF1, USF2, or a dominant negative (DN) form of USF1. When USFs were cotransfected together, only 1.5 µg of each expression vector was used. Promoter activity is expressed as fold induction, relative to the activity observed in the presence of empty expression vector. (B) The *FOXE1* promoter containing the A allele was cotransfected into HeLa cells with empty expression vectors or the same vector harbouring the cDNAs for α-CREM, CREB, USF1, USF2, or DREAM in the combinations shown in the figure. Numbers in the left hand panel represent µg of expression vector transfected; in the right hand panel, 3 µg of the indicated transcription factors were cotransfected. Promoter activity is expressed as fold induction, relative to the activity observed in the presence of empty (left) or α-CREM (right) expression vectors. Luciferase activity was normalized to Renilla activity derived from the cotransfected pRL-Tk vector to adjust for transfection efficiency. Results are mean±SD of four independent experiments. (*):*P*<0.05; (**):*P* = 0.01–0.001; (***):*P*<0.001; ns, not significant.

To validate the functional significance of the transcription factors identified for *FOXE1* gene expression, Hela cells were transiently transfected with expression vectors harbouring the cDNAs of the different transcription factors, together with the *FOXE1* reporter construct containing the rs1867277 A allele (pGl3b-FOXE1-283A). While DREAM transfection did not generate significant variations in *FOXE1* promoter activity, USF1 and USF2 transfection increased this activity (*P*<0.01 and *P*<0.05, respectively) (Figure 4A). Since USF homo- and heterodimer formation has been described [39], we cotransfected both expression vectors simultaneously, and observed a large increase (8-fold, *P*<0.0001) of *FOXE1* promoter activity. This effect was specific, since it was abolished when a dominant negative form of USF was cotransfected. Moreover, cotransfection of the DREAM expression vector together with USF1/USF2 clearly reduced the activation of the *FOXE1* reporter when compared to the USF1/USF2 condition (*P*<0.0001), confirming the corepressor function of DREAM [31],[40]. All these data highlight the relevant regulatory role of USF factors in the expression of *FOXE1*, as well as the inhibitory effect of DREAM on USF-dependent *FOXE1* activation. To verify that the effect of the USF factors is mediated through the previously mentioned putative USF binding site in the A allele, transient transfection assays were performed using pGl3b-FOXE1-283G, the reporter construct containing the G allele. No effect was found for USF1 and USF2 when used independently, and simultaneous cotransfection of USF1/2 resulted in a transcriptional activation that was clearly less than that observed with the A allele (*P*<0.0001). In summary, the rs1867277 G allele partially impairs the recruitment of USF1/2 factors to the *FOXE1* promoter and alters the expression status of the *FOXE1* gene.

### αCREM and CREB isoforms activate *FOXE1* transcription through a CRE site located close to rs1867277

Transcriptional activation of the *FOXE1* gene is regulated by hormonal factors, particularly by TSH via cAMP [41]. The transcription factors αCREM and CREB bind to CRE consensus sequences within the promoters of genes regulated by cAMP. Given the existence of a CRE-like site located near rs1867277 (Figure 2), we evaluated the role of αCREM and CREB in FOXE1 regulation. Overexpression of the two isoforms induced significant increases of *FOXE1* promoter activity, with αCREM being the most potent activating factor (changes >50 fold) (Figure 4B). In order to evaluate how this αCREM-dependent activation could be modified by the transcription factors that bind within the *FOXE1* promoter at the region containing the rs1867277 risk variant, various amounts of the USF1, USF2, CREB and DREAM expression vectors were transfected alone or in combination with fixed amounts of αCREM expression vector and FOXE1 reporter. USF1/USF2 and CREB did not affect αCREM-induced *FOXE1* reporter activity, while DREAM overexpression led to an 80% repression (*P*<0.0001). Considering these data, CRE-binding factors are acting as positive regulators of *FOXE1* gene expression, while DREAM displays a negative regulatory effect on αCREM-dependent regulation. Moreover, USF factors do not modulate the transcriptional activity of αCREM, which suggests the existence of two independent regulatory mechanisms that include DREAM and CRE-binding factors or USF proteins.

## Discussion

In the present work, by means of a two-step candidate-gene association study, we have identified *FOXE1* as a low penetrance gene (LPG) associated with papillary thyroid cancer (PTC). Regression analysis allowed us to pinpoint several SNPs within the *FOXE1* locus as highly significant risk-conferring variants for PTC. Haplotype analysis, followed by an exhaustive search within the sequence of the LD block containing the *FOXE1* gene, enabled us to identify a promoter SNP (rs1867277; c.−283 G>A) that we postulate to be a causal variant due to its predicted effect on a transcription factor binding site. The association results for this variant were validated in an independent population. The combined OR [per allele] was 1.49 (95% CI = 1.30–1.70; *P* = 5.9×10^−9^). Functional assays revealed that the c.−283A allele led to a differential recruitment of USF1 and USF2 transcription factors.

Thyroid cancer is believed to be a complex disease, in which common genetic variants located in low penetrance genes may interact with each other and with the environment, determining individual susceptibility. Our association results suggest that *FOXE1* is especially important for developing the classic PTC subtype, which represents around half of the PTC cases. This observation provides a basis for the heterogeneity described for this neoplasia, which includes more than 15 histological subtypes. Therefore, we propose that *FOXE1* is acting as an LPG related to thyroid cancer, which is in agreement with the increasing evidence of forkhead box (Fox) proteins having a crucial role in the development and progression of cancer, and their emerging role as potential biomarkers [42].

The FoxE1 transcription factor belongs to the forkhead family of transcription factors. These factors share a highly conserved winged helix DNA binding domain that is able to interact with nucleosomes and alter chromatin structure, creating a local exposed domain necessary for the action of other transcription factors. This property defined FOXE1 as a pioneer transcription factor [43], whose action is essential for the development, differentiation, and hormone responsiveness of the thyroid gland [22]. Thus, the control of its expression must be exquisitely regulated. However, few data are available concerning the transcription factors involved in *FOXE1* expression in the thyroid, although the fact that TSH controls *FOXE1* expression through cAMP and Ca^2+^ [41],[44] suggests that CRE- and Ca^2+^-binding factors may play a key role. In fact, sequence analysis of the *FOXE1* promoter, where rs1867277 (c.−283G>A) is located, revealed the existence of a DRE site, similar to the one previously found by D'Andrea et al. [45] and that shared a high similarity with a previously defined consensus DRE site in the prodynorphin gene [37]. Unexpectedly, functional assays showed that the Ca^2+^-dependent transcription factor DREAM bound equally well to both rs1867277 alleles.

The most remarkable functional data obtained was the formation of a DNA-protein complex exclusively between the A allele and USF1/USF2 factors. These are ubiquitously expressed proteins, which belong to the basic helix-loop-helix (HLH) leucine zipper family of transcription factors. They share a highly conserved C-terminal domain responsible for dimerization and DNA binding, which recognizes the canonical E-box sequence CACGTG [39],[46]. The involvement of USF factors in *FOXE1* regulation was confirmed by a transfection approach. These factors, which mainly act as heterodimers, induced significant increases in *FOXE1* transcriptional activity when the rs1867277 A allele was present. These data agree both with the role of USF factors as positive transcriptional regulators of their target genes [47] and with the predominant role of USF1/USF2 heterodimers in comparison with homodimers [39].

Moreover, interactions between N-terminal domains of USF dimers and cell-specific transcription factors have been described to be involved in cooperative transcriptional regulation [48]. Interestingly, we identified a potential CRE site located near the rs1867277 sequence, which could act as a target of CRE-related proteins (CREB and αCREM). Transient transfection assays demonstrated that CREB, and more strongly αCREM, activate the *FOXE1* promoter, while DREAM reduces significantly αCREM dependent-transcriptional induction. These data suggest a direct competition between CRE-binding factors and DREAM for binding to the *FOXE1* promoter, which will ultimately control the transcriptional status of the *FOXE1* gene. In fact, it has been described that Ca2+ and cAMP concentrations modulate a regulatory network which links CRE-binding proteins and DREAM [38],[49]. In this way, these transcription factors could regulate the expression of *FOXE1* in response to TSH in a physiological situation.

Given that both CRE proteins and USF factors are associated with an increase in *FOXE1* expression levels through closely neighbouring sites, the question arises if these factors also cooperate to regulate *FOXE1* transcription. Our results showed no synergistic effect of αCREM and USF1/2 proteins on *FOXE1* transcription, which is in agreement with other reports [50],[51]. It therefore remains to be elucidated which additional proteins, if any, are acting together with USF proteins in modulating *FOXE1* expression.

Finally, considering the specific binding of USF factors to the disease risk-conferring rs1867277 A allele, an increased expression of *FOXE1* in thyroid follicular cell tumours in comparison to normal thyrocytes is to be expected. Few data are available regarding *FOXE1* status in thyroid cancer, although Sequeira *et al* demonstrated that an increased *FOXE1* expression paralleled the dedifferentiation process of thyroid carcinomas [52]. Therefore, it is necessary to understand in which manner FOXE1, a thyroid transcription factor involved in the maintenance of the differentiated adult thyroid phenotype, could be involved in acquiring a malignant status. One possible explanation relies on the results obtained from *FOXE1* knockout mouse models. During embryonic development, thyroid cell precursors require *FOXE1* transcription initially to allow their own migration from the thyroid bud, and later to constitute a functional endocrine organ [21],[53]. Considering these data, we hypothesised that increased *FOXE1* expression in thyroid carcinomas could be related to a motile advantage of malignant thyroid cells, which would be enhanced by the presence of the rs1867277 A risk predisposing allele. While FOXE1-specific studies are needed to further understanding the role of this gene in thyroid tumour cell migration and invasion, several studies have confirmed a tumoral role of forkhead family of transcriptional factors. In this regard, genes encoding forkhead factors, among others, have been recently identified as a molecular signature for Epithelial to Mesenchymal transition in a human colon cancer [54] and overexpression of FOX factors has been described in several cancers [55]–[62]. This opens an interesting future for understanding the role of *FOXE1* in thyroid tumour cell migration and invasion.

Taken together, our association study, combined with a functional assessment, allowed us to pinpoint a causal variant within the *FOXE1* promoter, and to propose a mechanism by which this causal variant acts as a genetic risk factor specifically related to PTC susceptibility. This finding reveals the importance of considering a complex disease such as thyroid cancer as a heterogeneous entity. It is crucial not to study complex diseases as a collective, but to cluster cases according to homogeneous and well-established clinical features. It seems obvious that just one variant cannot explain the phenotype, and many other signals and mechanisms may be also involved. However, our results are an important step forward in understanding the disease, offering new insights into the genetic mechanism involved in non-medullary thyroid cancer development. It is also important to remember that the complex nature of each locus could be operative through additional variants or mechanisms.

In addition, our study validates the results of the first GWAS performed in thyroid cancer [23], and addresses one of the major limitations of GWAS: the enormous difficulty to provide a link between a significant intergenic tagSNP and the precise variant that has a causal role and provides a biological explanation [63]. The modest power to identify causal variants using GWAS is in part because, in this hypothesis-free approach, approximately 20% of common SNPs are partially tagged, and rare variants are not tagged at all. Overall, our study provides proof that, when reliable biological knowledge or expression data from the tissues of interest are available, candidate gene approaches can be straightforward to identify low penetrance genes and to find putative causal variants within those genes.

## Supporting Information

Figure S1 Layout of the two-step association study. The diagram summarizes our two-step association approach, which initially included a Spanish series (Phase I) of 615 cases and 525 controls. Seventy-eight additional Spanish cases were subsequently included and analysed by KASPar platform. Phase II, composed of 482 cases and 532 Italian controls, was used as a validation set. Top association results were obtained for the functional variant rs1867277 (shown in blue), within *FOXE1* gene. *P* values (also shown in Table 3) correspond to papillary thyroid cancer (PTC) cases *versus* controls. Small boxes indicate the genotyping platform, the mean age, and the female:male ratio (matched in cases and controls) of each group.
(2.04 MB TIF)

Table S1 Association results for *FOXE1* SNPs in the Spanish population (Phase I) after stratification by subtype.
(0.04 MB DOC)

Table S2 Group-specific associated *P* values obtained using the PHASE program for the haplotype distribution between Spanish cases and controls.
(0.03 MB DOC)

Table S3 Pairwise Linkage Disequilibrium values, expressed as D' parameter, for the Illumina tested SNPs within the FOXE1 LD block, the functional variant rs1867277, and the top associated SNP rs965513 by Gudmundsson *et al*.
(0.05 MB DOC)
