# Supplementary figures and images for: The Variant rs1867277 in FOXE1 Gene Confers Thyroid Cancer Susceptibility through the Recruitment of USF1/USF2 Transcription Factors
Source: PLoS Genet. 2009 Sep 4;5(9):e1000637. doi: 10.1371/journal.pgen.1000637 (PMC2727793; doi:10.1371/journal.pgen.1000637)

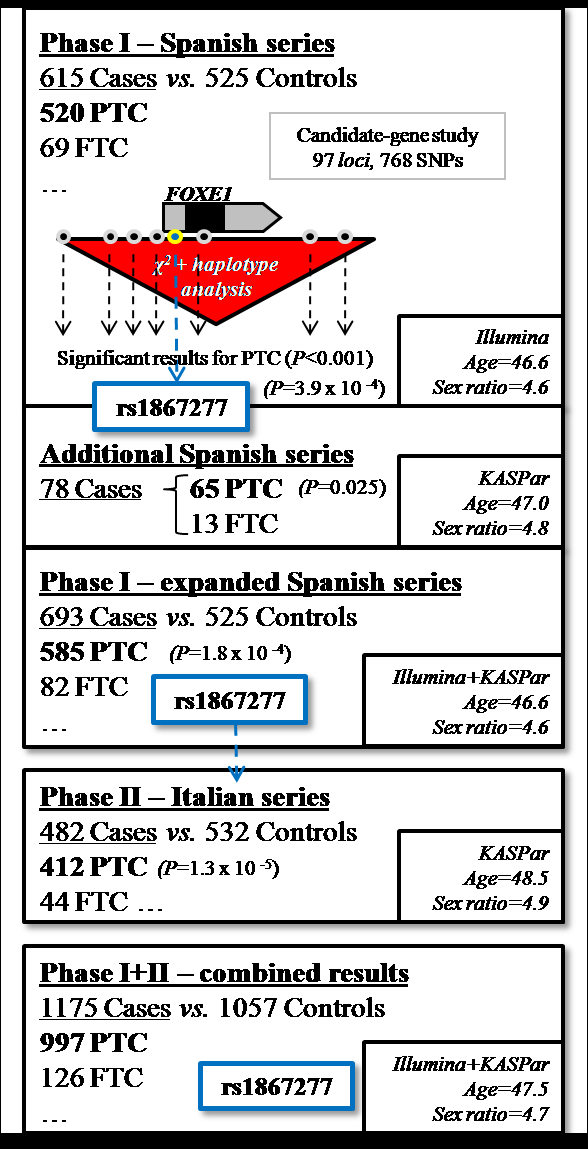

Supplement: Figure S1 — Layout of the two-step association study. The diagram summarizes our two-step association approach, which initially included a Spanish series (Phase I) of 615 cases and 525 controls. Seventy-eight additional Spanish cases were subsequently included and analysed by KASPar platform. Phase II, composed of 482 cases and 532 Italian controls, was used as a validation set. Top association results were obtained for the functional variant rs1867277 (shown in blue), within FOXE1 gene. P values (also shown in Table 3) correspond to papillary thyroid cancer (PTC) cases versus controls. Small boxes indicate the genotyping platform, the mean age, and the female:male ratio (matched in cases and controls) of each group. (2.04 MB TIF) [file pgen.1000637.s001.tif]
