# Supplementary material for: The Variant rs1867277 in FOXE1 Gene Confers Thyroid Cancer Susceptibility through the Recruitment of USF1/USF2 Transcription Factors
Source: PLoS Genet. 2009 Sep 4;5(9):e1000637. doi: 10.1371/journal.pgen.1000637 (PMC2727793; doi:10.1371/journal.pgen.1000637)
Supplement: Table S1 — Association results for FOXE1 SNPs in the Spanish population (Phase I) after stratification by subtype. (0.04 MB DOC) [file pgen.1000637.s002.doc]

**Supporting Information**

**Table S1. Association results for *FOXE1* SNPs in the Spanish population (Phase I) after stratification by subtype**

|  | | **Controls** | | **classic PTC (cPTC)** | | | | **Follicular Variant of PTC (FVPTC)** | | | | **Follicular Thyroid Carcinoma (FTC)** | | | |
| --- | --- | --- | --- | --- | --- | --- | --- | --- | --- | --- | --- | --- | --- | --- | --- |
| **SNP ID** | **Alleles** | **n** | **MAF** | **n** | **MAF** | **OR (95%CI)** | ***P*** | **n** | **MAF** | **OR (95%CI)** | ***P*** | **n** | **MAF** | **OR (95%CI)** | ***P*** |
| rs7048394 | C/T | 520 | 0.243 | 304 | 0.329 | 1.56 (1.24-1.96) | **1.6 x 10^-4^** | 146 | 0.271 | 1.16 (0.86-1.58) | 0.328 ns | 69 | 0.275 | 1.19 (0.79-1.79) | 0.403 ns |
| rs894673 | T/A | 520 | 0.400 | 304 | 0.493 | 1.45 (1.19-1.78) | **3.2 x 10^-4^** | 146 | 0.442 | 1.18 (0.91-1.53) | 0.208 ns | 69 | 0.413 | 1.06 (0.74-1.51) | 0.770 ns |
| rs3758249 | G/A | 517 | 0.399 | 303 | 0.493 | 1.46 (1.19-1.78) | **2.7 x 10^-4^** | 144 | 0.441 | 1.18 (0.91-1.53) | 0.207 ns | 69 | 0.413 | 1.06 (0.74-1.51) | 0.749 ns |
| rs907577 | A/G | 518 | 0.399 | 303 | 0.491 | 1.45 (1.18-1.78) | **3.4 x 10^-4^** | 145 | 0.441 | 1.19 (0.91-1.54) | 0.201 ns | 69 | 0.413 | 1.06 (0.74-1.51) | 0.749 ns |
| rs3021526 | T/C | 475 | 0.398 | 281 | 0.479 | 1.38 (1.12-1.96) | **2.5 x 10^-3^** | 128 | 0.437 | 1.17 (0.89-1.53) | 0.264 ns | 68 | 0.404 | 1.03 (0.72-1.47) | 0.888 ns |
| rs10119760 | C/G | 518 | 0.344 | 302 | 0.454 | 1.57 (1.27-1.93) | **2.0 x 10^-5^** | 146 | 0.387 | 1.19 (0.92-1.55) | 0.189 ns | 69 | 0.384 | 1.18 (0.82-1.69) | 0.369 ns |
| OR [per allele] obtained by comparing cases against controls and considering the minor allele as the risk allele. Abbreviations: MAF=Minor Allele Frequency; OR=Odds Ratio; CI=Confidence Interval; ns=not significant | | | | | | | | | | | | | | | |
