# Supplementary material for: The Variant rs1867277 in FOXE1 Gene Confers Thyroid Cancer Susceptibility through the Recruitment of USF1/USF2 Transcription Factors
Source: PLoS Genet. 2009 Sep 4;5(9):e1000637. doi: 10.1371/journal.pgen.1000637 (PMC2727793; doi:10.1371/journal.pgen.1000637)
Supplement: Table S2 — Group-specific associated P values obtained using the PHASE program for the haplotype distribution between Spanish cases and controls. (0.03 MB DOC) [file pgen.1000637.s003.doc]

**Supporting Information**

**Table S2.** Group-specific associated *P* values obtained using the PHASE program for the haplotype distribution between Spanish cases and controls

| **Group** | **Cases (n)** | **PHASE associated *P*** |
| --- | --- | --- |
| All PTC | 520 | **0.0147** |
| cPTC | 305 | **0.0005** |
| FVPTC | 146 | 0.8914 |
| FTC | 69 | 0.3322 |

* The LD region studied spans from coordinates 99,648,503 and 99,668,059 on chromosome 9 for a total length of 19.5 kb. Polymorphisms tagging the region were rs894673, rs3758249, rs907577, rs1867277, rs3021526, rs874004, and rs10119760. All results refer to haplotype distribution of cases against the control group (n=504). Abbreviations: PTC=Papillary Thyroid Carcinoma; cPTC=classic PTC; FVPTC= follicular variant of PTC; FTC=Follicular Thyroid Carcinoma.
