# Supplementary material for: The Variant rs1867277 in FOXE1 Gene Confers Thyroid Cancer Susceptibility through the Recruitment of USF1/USF2 Transcription Factors
Source: PLoS Genet. 2009 Sep 4;5(9):e1000637. doi: 10.1371/journal.pgen.1000637 (PMC2727793; doi:10.1371/journal.pgen.1000637)
Supplement: Table S3 — Pairwise Linkage Disequilibrium values, expressed as D' parameter, for the Illumina tested SNPs within the FOXE1 LD block, the functional variant rs1867277, and the top associated SNP rs965513 by Gudmundsson et al. (0.05 MB DOC) [file pgen.1000637.s004.doc]

**Supporting Information**

**Table S3. Pairwise Linkage Disequilibrium values, expressed as D' parameter, for the Illumina tested SNPs within the FOXE1 LD block, the functional variant rs1867277 and the top associated SNP rs965513 by Gudmundsson *et al*.**

| **Dist (Kb) ***** | **-59,8** | **-3,6** | **-1,8** | **-0,8** | **0** | **+1,1** | **+6,2** | **+8,7** |
| --- | --- | --- | --- | --- | --- | --- | --- | --- |
| **SNP ID ** (coordinate)** | **rs965513 (99,595,930)** | **rs894673 (99,652,091)** | **rs3758249 (99,653,961)** | **rs907577 (99,654,938)** | **rs1867277 (99,655,735)** | **rs3021526 (99,656,842)** | **rs874004 (99,661,939)** | **rs10119760 (99,664,423)** |
|
| **rs965513 (99,595,930)** | **CEU/ESP** * | 0.74 / NA | 0.73 / NA | 0.73 / NA | 0.73 / NA | 0.89 / NA | 0.71 / NA | 0.62 / NA |
|
|  | **rs894673 (99,652,091)** |  | 1.00 / 1.00 | 1.00 / 1.00 | 1.00 / 1.00 | 1.00 / 1.00 | 1.00 / 0.98 | 0.96 / 0.99 |
|  |
|  |  | **rs3758249 (99,653,961)** |  | 1.00 / 1.00 | 1.00 / 1.00 | 1.00 / 1.00 | 1.00 / 0.98 | 0.96 / 0.99 |
|  |  |
|  |  |  | **rs907577 (99,654,938)** |  | 1.00 / 1.00 | 1.00 / 1.00 | 1.00 / 0.99 | 0.96 / 0.99 |
|  |  |  |
|  |  |  |  | **rs1867277 (99,655,735)** |  | 1.00 / 1.00 | 1.00 / 0.99 | 0.96 / 0.99 |
|  |  |  |  |
|  |  |  |  |  | **rs3021526 (99,656,842)** |  | 1.00 / 0.99 | 0.93 / 0.99 |
|  |  |  |  |  |
|  |  |  |  |  |  | **rs874004 (99,661,939)** |  | 1.00 / 1.00 |
|  |  |  |  |  |  |
|  |  |  |  |  |  |  | **rs10119760 (99,664,423)** |  |
|  |  |  |  |  |  |  |

* D' values are calculated from both HapMap-CEU population (CEU) and our Spanish control series (ESP). D' values for rs1867277 and rs3021526 were not available in databases and were inferred by their correlated SNPs rs907577 and rs3021523, respectively. NA=not assessed for this population. ** The functional variant rs1867277 is highlighted in blue, and its neighboring fully correlated tagSNP is displayed in red. The top association identified by Gudmundsson et al is shown in green. *** Physical distance is measured as kilobases from the functional variant rs1867277.
